# A case of preoperative embolization for a giant hypervascular pancreatic serous cystic neoplasm in pancreaticoduodenectomy

**DOI:** 10.1186/s40792-024-02009-2

**Published:** 2024-09-06

**Authors:** Takahito Matsuyoshi, Naoki Ikenaga, Kohei Nakata, Daisuke Okamoto, Takashi Matsumoto, Toshiya Abe, Yusuke Watanabe, Noboru Ideno, Keizo Kaku, Nao Fujimori, Kenoki Ohuchida, Yasuhiro Okabe, Yoshinao Oda, Kousei Ishigami, Masafumi Nakamura

**Affiliations:** 1 Department of Surgery and Oncology, Graduate School of Medical Sciences, Kyushu University, 3-1-1 Maidashi, Higashi-ku, Fukuoka, 812-8582 Japan; 2 Department of Clinical Radiology, Graduate School of Medical Sciences, Kyushu University, Maidashi, Fukuoka, Japan; 3 Department of Anatomic Pathology, Pathological Sciences, Graduate School of Medical Sciences, Kyushu Hospital, Fukuoka, 812-8582 Japan; 4 Department of Medicine and Bioregulatory Science, Graduate School of Medical Sciences, Kyushu University, Maidashi, Fukuoka, Japan

**Keywords:** Serous cystic neoplasm, Pancreaticoduodenectomy, Bleeding, Hemorrhage, Preoperative embolization, Interventional radiology

## Abstract

**Background:**

Preoperative vascular embolization is an effective strategy for managing meningiomas, neck paragangliomas, renal cell carcinomas, and bone metastasis by reducing the intraoperative bleeding volume and operation time. Although hypervascular tumors also occur in the pancreas, preoperative embolization for these tumors is not commonly practiced. We herein present a case of a giant serous cystic neoplasm (SCN) of the pancreas with significant arterial vascularity that was managed with preoperative interventional radiology and subsequently resected via pancreaticoduodenectomy.

**Case presentation:**

A 60-year-old man presented with an 8-cm hypervascular tumor located at the head of the pancreas, identified as an SCN on pathologic examination. The tumor had increased by 13 mm over 5 years, necessitating surgical intervention. Computed tomography revealed a substantial blood supply to the tumor from the dorsal pancreatic artery and gastroduodenal artery, both branches of the superior mesenteric artery. To mitigate the risk of severe intraoperative bleeding from this giant hypervascular tumor, branches of the dorsal pancreatic artery and gastroduodenal artery were embolized using metallic coils and further secured using a gelatin sponge 1 day prior to pancreatectomy. During the laparotomy, the tumor appeared to have decreased in size, likely because of reduced distension and congestion. Despite significant adhesions to surrounding tissues secondary to prolonged compression and inflammation, the pancreaticoduodenectomy was completed successfully in 5 h and 15 min with blood loss of 763 mL. The patient was discharged on postoperative day 15 without complications.

**Conclusions:**

Preoperative arterial embolization for hypervascular pancreatic tumors might control the risk of massive intraoperative bleeding, contributing to a favorable postoperative outcome. Utilizing interventional radiology for preoperative inflow control is one of the beneficial strategies for pancreatectomy in patients with a giant SCN.

## Background

High tumor vascularity can increase the risk of significant intraoperative bleeding, leading to prolonged operation times and increased patient morbidity [1, 2]. In hypervascular tumors such as meningiomas [3], neck paragangliomas [2, 4], renal cell carcinomas [5, 6], and metastases to the spine and long bones from primary thyroid and renal cell carcinomas [7, 8], preoperative vascular embolization has been shown to reduce intraoperative bleeding, the need for blood transfusions, and the operation time, thereby enhancing surgical safety. Although the pancreas also develops hypervascular tumors, including serous cystic neoplasms (SCNs) and neuroendocrine tumors, preoperative vascular embolization is not routinely practiced in pancreatic surgeries.

SCNs are benign tumors characterized by clusters of tiny cysts, and the septa of these cysts contain abundant blood vessels. The risk of malignancy is exceptionally low [9]. However, resection is recommended for tumors exceeding 4 cm in size or those that are rapidly growing because of potential for malignancy or symptomatic manifestations, such as abdominal pain, fullness, and jaundice [10, 11]. SCNs can occasionally grow extremely large and develop severe inflammatory adhesions to surrounding tissues, complicating surgical manipulation and dissection [12].

In this report, we present a case of pancreatic resection of a giant hypervascular SCN. Preoperative embolization of the tumor’s feeding vessels facilitated safe pancreaticoduodenectomy. We discuss the strategy and clinical significance of preoperative vascular embolization for managing hypervascular pancreatic tumors.

## Case presentation

**Fig. 1:**
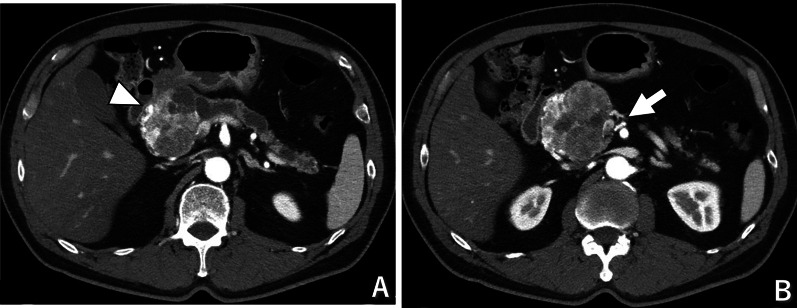
Enhanced computed tomography findings. **A** An 8-cm tumor was located at the head of the pancreas, composed of numerous tiny cysts with abundant vascularization. The main pancreatic duct was obstructed by the tumor, and the distal pancreatic duct was dilated. The arrowhead shows the gastroduodenal artery, a feeding artery of the tumor. **B** The dorsal pancreatic artery, branching from the superior mesenteric artery, was another feeder of the tumor (arrow)

A 60-year-old man with a history of hypertension and diabetes mellitus presented with an incidentally discovered pancreatic tumor during an evaluation for acute appendicitis 5 years prior. The tumor was diagnosed as an SCN following pathologic examination using ultrasound-guided tissue acquisition, and the patient underwent annual monitoring by his family physician. Over the 5-year period, the tumor had enlarged by 13 mm, prompting a referral to our department. Computed tomography revealed an 8-cm tumor located at the head of the pancreas, characterized by a microcystic structure and significant intra-tumor vascular proliferation (Fig. 1A). The primary vascular supply to the tumor included the gastroduodenal artery (GDA) and dorsal pancreatic artery (DPA), both branching from the superior mesenteric artery (SMA) (Fig. 1B). The tumor caused obstruction of the main pancreatic duct at the head and resulted in atrophy of the pancreatic tail. Laboratory results indicated a mild increase in the carbohydrate antigen 19-9 concentration to 40.2 U/mL. Considering the tumor’s growth and potential for future symptomatic progression or malignancy, a decision was made to perform pancreaticoduodenectomy to excise the tumor.

**Fig. 2:**
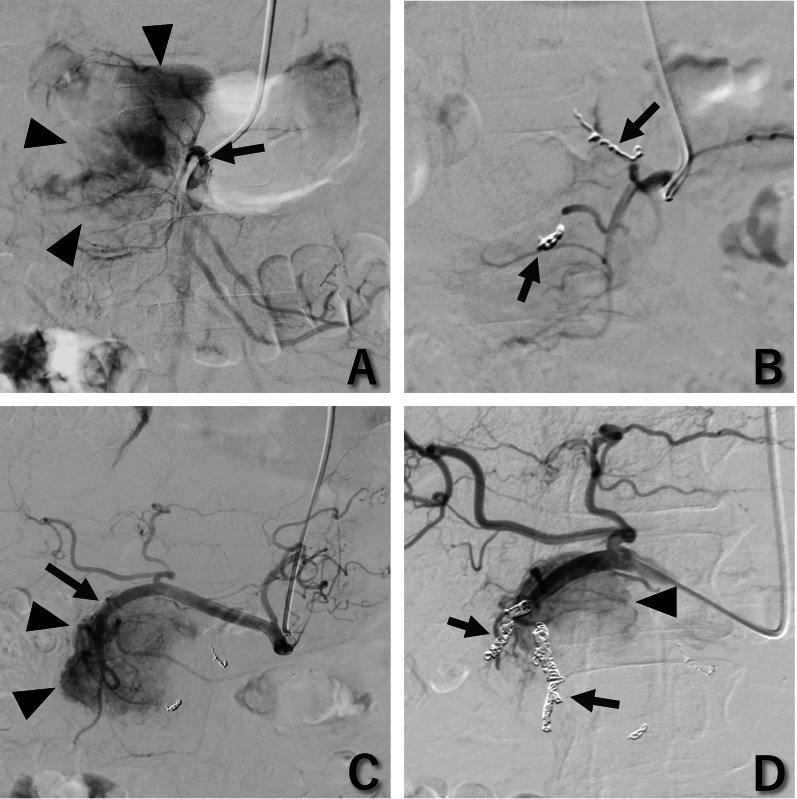
Interventional radiology findings. **A** A contrast injection from the superior mesenteric artery showed vascularization of the left side of the tumor (arrowheads) via the dorsal pancreatic artery (arrow). **B** Distal branches from the dorsal pancreatic artery were embolized with metallic coils (arrows) and subsequent application of a gelatin sponge, resulting in cessation of the tumor’s blood supply from the dorsal pancreatic artery. **C** A contrast injection from the celiac artery showed vascularization of the right side of the tumor (arrowheads) via the gastroduodenal artery (arrow). **D** Distal branches from the gastroduodenal artery were partially embolized with metallic coils (arrows) alone, leading to diminished vascularization of the tumor. The blood supply to the tumor remained (arrowhead)

To mitigate the risk of significant hemorrhage during surgical removal of the giant hypervascular tumor, we performed preoperative vascular embolization 1 day prior to the pancreatectomy after detecting feeding arteries via computed tomography. The primary embolization target was the DPA, located on the posterior aspect of the tumor, because it was more accessible through interventional radiology (IR) techniques than through surgical approaches. SMA angiography revealed the DPA as the main vascular supply to the tumor (Fig. 2A). In the DPA, metallic coils alone did not completely interrupt the blood flow, and we were concerned that further addition of coils might interfere with vessel ligation during the pancreaticoduodenectomy. Therefore, a gelatin sponge was used to halt the blood flow from this artery (Fig. 2B). Further angiographic examination of the celiac axis revealed additional tumor feeders from the anterior superior pancreaticoduodenal artery and posterior superior pancreaticoduodenal artery (Fig. 2C). These arteries were partially occluded using only coils to reduce the blood supply to the tumor (Fig. 2D). The gelatin sheet was typically hand-cut into pieces measuring 1 to 2 mm in size [13]. Following embolization, the patient remained asymptomatic with normal laboratory indices.

**Fig. 3:**
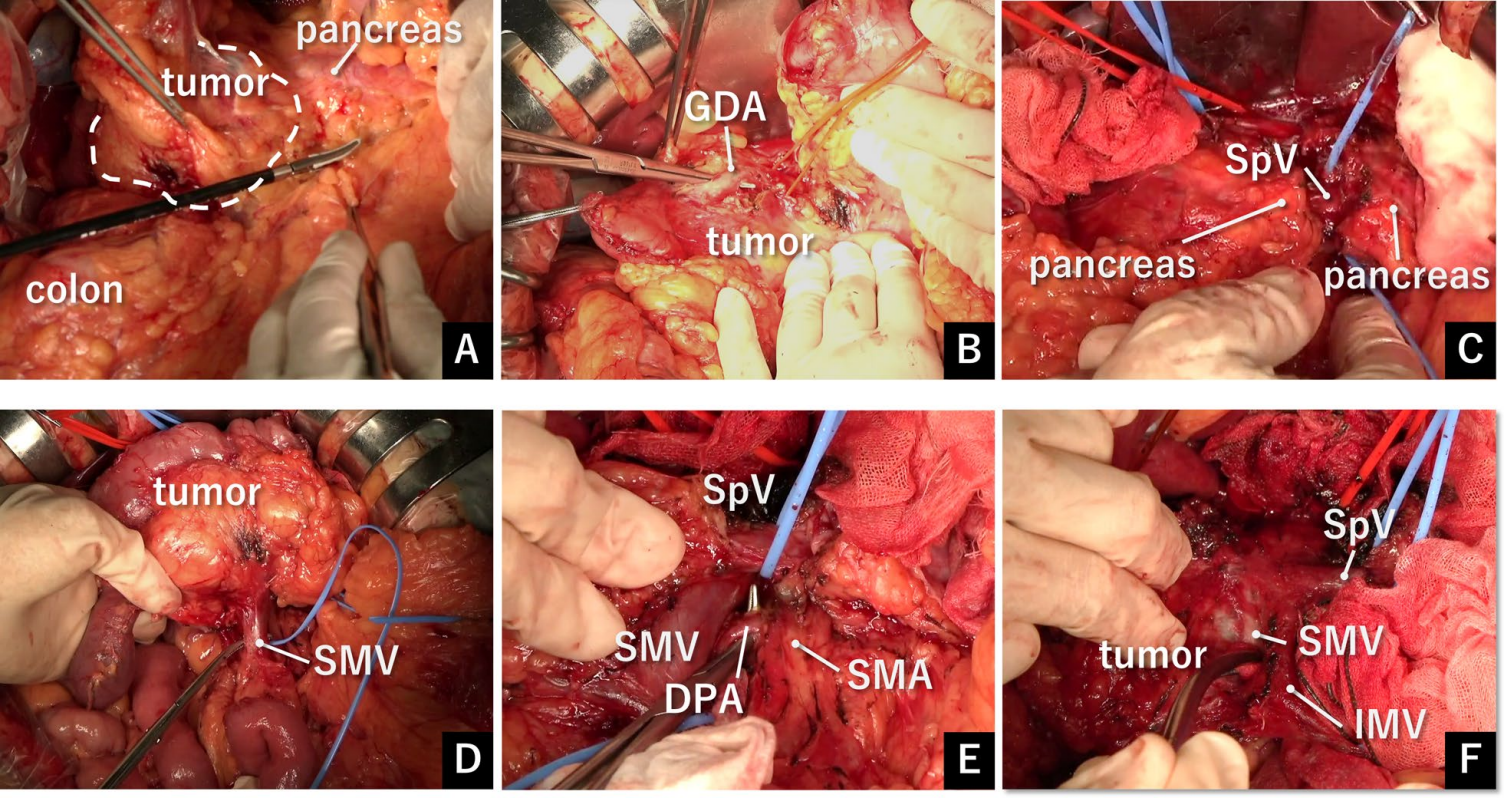
Operative findings. **A** The large tumor was severely adhered to surrounding tissues, including the stomach and transverse mesocolon. No tumor distension or congestion was observed following preoperative arterial embolization. **B** The GDA, which was adhered to the tumor surface, was ligated at the start of the surgery to completely halt the blood supply to the tumor. **C** The pancreas was divided above the superior mesenteric artery, exposing the SpV. **D** The SMV was secured with tape as a precaution against potential massive bleeding during tumor dissection from the SMV. **E** The DPA, branching from the SMA, was identified using an anterior approach. **F** The tumor was strongly adhered to the SMV as a result of long-standing inflammation. *GDA* gastroduodenal artery, *SpV* splenic vein, *SMV* superior mesenteric vein, *SMA* superior mesenteric artery, *DPA* dorsal pancreatic artery, *IMV* inferior mesenteric vein

**Fig. 4:**
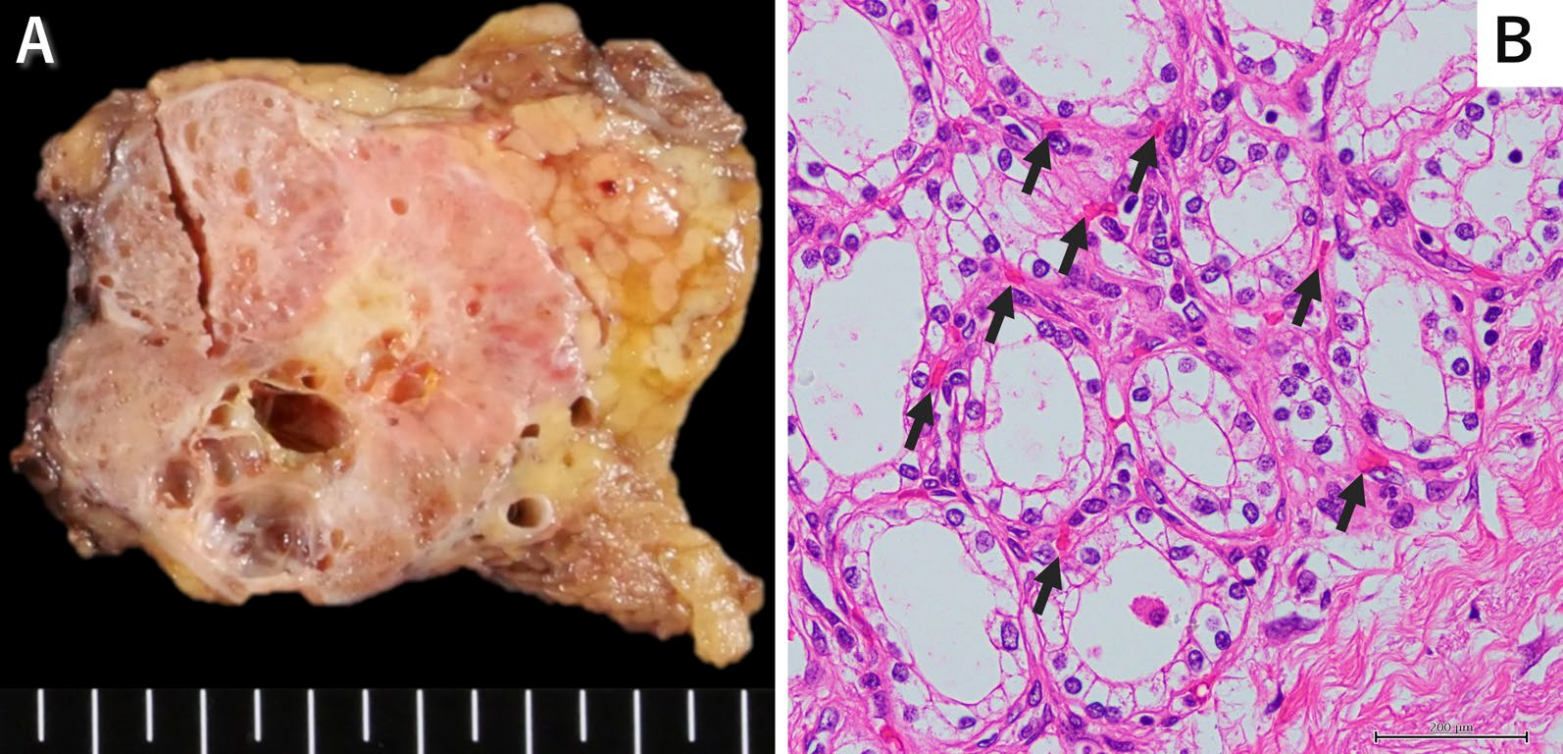
Histopathological findings. **A** Macroscopically, the microcystic tumor was located on the pancreas head with no evidence of bleeding or necrosis. The tumor measured 68 × 43 × 41 mm. **B** Microscopically, the cyst wall was lined by a single layer of cuboidal epithelium without atypia. Red blood cells (arrows) were among the epithelial cells. Hematoxylin and eosin, ×400

Pancreaticoduodenectomy was performed the subsequent day. The tumor measured 8 cm and was tightly adhered to surrounding structures, including the stomach, transverse mesocolon, and common hepatic artery, consistent with ongoing obstructive pancreatitis (Fig. 3A). Notably, the superior mesenteric vein (SMV) was positioned posterior to the large tumor and tightly adhered, complicating pancreatic tunneling over the SMV. However, tumor distension and congestion were absent, likely because of arterial embolization, indicating a reduction in tumor volume (Fig. 3A). Early in the surgery, the GDA, superficial to the tumor, was ligated to completely block the blood supply to the tumor (Fig. 3B). The pancreas was then transected above the SMA, and the tumor was dissected away from the splenic vein toward the pancreatic head (Fig. 3C). Prior to dissecting the tumor from the SMV, both the SMV and portal vein were secured with tape to prevent excessive bleeding (Fig. 3D). The origin of the DPA from the SMA was exposed via an anterior approach and subsequently ligated (Fig. 3E). The tumor was finally separated from the SMV and portal vein (Fig. 3F). During this process, the posterior superior pancreaticoduodenal vein was damaged at the confluence with the portal vein because of the tumor’s firm adhesion, accounting for the majority of the estimated blood loss during surgery. We controlled the hemorrhage by suturing the portal vein while temporarily occluding it, completing the pancreaticoduodenectomy. The operation lasted 5 h and 15 min, and the total blood loss of 763 mL was managed without transfusion. The patient’s postoperative course was uneventful, and he was discharged on postoperative day 15. Histopathologic examination confirmed the diagnosis of a microcystic adenoma subtype of SCN, measuring 68 × 43 × 41 mm (Fig. 4A). Microscopically, the cyst wall was lined by a single layer of cuboidal epithelium with no malignant features (Fig. 4B). The tumor also exhibited a rich capillary network, as evidenced by numerous red blood cells dispersed among the epithelial cells, consistent with a previous report [14].

## Discussion

Surgical interventions for hypervascular tumors pose significant bleeding risks as a result of the tumors’ high vascularity. Consequently, preoperative embolization has become a practical strategy for managing some solid tumors. Preoperative embolization is known to reduce intraoperative bleeding, transfusion requirements, and operation time in conditions such as meningiomas [3], neck paragangliomas [2, 4], renal cell carcinomas [5, 6], and metastases to the spine and long bones from primary thyroid and renal cell carcinomas [7, 8]. For neck paragangliomas, embolization prior to surgery reportedly facilitates precise dissection around nerves, thus minimizing postoperative hypoglossal nerve paralysis [15]. Enhanced surgical safety from reduced bleeding not only ameliorates the immediate postoperative course, but also enhances the patient’s quality of life. In the present case, preoperative embolization for a giant pancreatic tumor with substantial blood flow may have controlled the intraoperative bleeding, promoting an uncomplicated postoperative recovery. Embolization was performed 1 day before the surgery because surgical resection is recommended within 48 h following vascular embolization to avoid complications associated with collateral vessel formation seen in other tumors [3, 16]. The selective embolization of feeding arteries 1 day before surgery did not lead to any symptomatic complications. As with other hypervascular tumors, preoperative embolization of giant pancreatic tumors with notably high blood flow might be an effective strategy.

SCNs are characterized by a very low malignant potential [9]. Surgical resection is considered for tumors exceeding 4 cm, exhibiting suspicious features of malignancy, exhibiting rapid growth (> 4 mm annually), or causing symptoms [10, 11]. Giant SCNs can induce severe inflammation and adhesions, compressing multiple surrounding organs and tissues and thus complicating surgical interventions. In some cases, the resection of adjacent organs and major vessels, such as the colon and portal vein, is necessary for the removal of large SCNs [12, 17–19]. Liu et al. [12] reported six surgical cases of giant SCNs. In one instance, pancreaticoduodenectomy resulted in incomplete resection because of extensive involvement of the portal vein, necessitating intraoperative repair. Another case required partial resection of the transverse colon given the tumor’s firm adhesions [12]. Furthermore, giant SCNs often develop numerous arterial feeders, leading to the expansion of fragile venous structures and complicating pancreatectomy. The tumor in the present case had two primary feeding arteries: the GDA, which was partially embolized, and the DPA, which was completely embolized. This approach effectively prevented tumor distension and reduced the size of enlarged draining veins, facilitating surgical management. Embolization of the DPA, which fed into the dorsal side of the tumor, proved crucial because this area was initially challenging to access. In addition, transecting the pancreas above the SMA line and detaching the tumor from the splenic vein toward the pancreatic head allowed for safe, complete resection, despite late injury to a portal vein branch. Preoperative embolization is one of the effective management strategies for giant SCNs, considering the complexities associated with pancreaticoduodenectomy for such tumors with high vascularity.

To date, three cases of vascular embolization for SCNs, including ours, have been reported (Table 1) [17, 20]. In one instance involving a tumor located at the pancreatic head [17], embolization was performed preoperatively to minimize intraoperative bleeding 1 day before surgery, similar to our procedure. Another case involved achieving hemostasis for bleeding from a large SCN at the pancreatic body/tail [20]. The selection of target arteries for embolization was based on tumor location. The feeding arteries of the GDA and DPA were embolized for the tumor at the pancreatic head, whereas the splenic artery and dorsal branch of the SMA were targeted for the tumor at the pancreatic body/tail. In all instances, the tumors were successfully removed post-embolization without complications during IR and surgery. Typically, arterial embolization in the pancreas addresses the rupture of pseudoaneurysms following acute pancreatitis, postoperative pancreatic fistulas, and median arcuate ligament syndrome. In previous cases [17, 20], vascular embolization was performed using metallic coils alone. In contrast, our approach combined metallic coils with subsequent placement of a gelatin sponge. The purpose of the gelatin sponge was to achieve complete occlusion of the DPA inflow without interfering with its ligation. The advantage of using a gelatin sponge is that it reduces blood flow to the tumor. The disadvantage, however, is the risk of complications associated with backflow into untargeted vessels, such as pancreatitis, pancreatic infarction, ileal ischemia, and abscesses in the liver and spleen. To minimize the risk of such complications, the sponge was placed selectively and in minimal quantities to the targeted vessels [21, 22]. Considering the potential complications, the decision to use preoperative embolization for controlling intraoperative bleeding requires a careful evaluation of the risks and benefits. In our case, the tumor was situated at the pancreatic head and had significant adhesions to the portal vein, necessitating extensive surgery. Notably, pancreatic infarctions occur less frequently with embolization at the head of the pancreas than at the tail [21]. Additionally, super-selective catheterization can further reduce the risk of complications [22]. We believe our method of selective arterial embolization utilizing mechanical occlusion devices and particulates provides clinical advantages that outweigh the associated risks of IR.

**Table 1:** Previously published case reports of embolization for serious cystic neoplasms

Case	Tumor size (cm)	Tumor location	Purpose of embolization	Embolized vessels	Interval between embolization and operation	Embolization agents
Tajima et al. [15]	13	Head	Reduce intraoperative bleeding	GDA, DPA, branch of SpA	1 day	Metallic coil
Amaral et al. [20]	15.5	Body/tail	Hemostasis	SpA, branch of SMA	3 weeks	Metallic coil
Present case	8	Head	Reduce intraoperative bleeding	DGA, DPA	1 day	Metallic coil, gelatin sponge

*SpA* splenic artery, *SMA* superior mesenteric artery, *GDA* gastroduodenal artery, *DPA* dorsal pancreatic artery

Currently, a hybrid operating room with on-table angiography allows for both inflow embolization and immediate surgery under the same anesthetic session in a single procedure [23]. This approach potentially prevents collateral inflow and reduces post embolization syndrome, including severe pain and fever [23]. In our case, however, the benefits of using a hybrid operating room with on-table angiography may have been limited. Although post-embolization syndrome is typically associated with inflammation and tissue necrosis, partial blood flow from the SMA remained preserved in our case. Consequently, we considered that post-embolization symptoms were unlikely to occur, and indeed, no adverse effects were observed. Additionally, the reduced image quality of angiography in the hybrid operating room approach can complicate the vascular embolization procedure. Thus, the effectiveness of this approach should be evaluated by analyzing additional cases.

## Conclusions

Preoperative arterial embolization for hypervascular pancreatic tumors might control the risk of massive intraoperative bleeding, potentially promoting a favorable postoperative course. Pancreaticoduodenectomy for a giant SCN can be challenging, given the difficulty in managing the tumor, its extensive vascularity, and severe inflammatory adhesions. Employing IR for preoperative inflow control is one of the valuable pancreatectomy strategies for large hypervascular pancreatic tumors.

## Data Availability

Not applicable.

## Abbreviations

SCN: Serous cystic neoplasm
IR: Interventional radiology
GDA: Gastroduodenal artery
DPA: Dorsal pancreatic artery
SMA: Superior mesenteric artery
SMV: Superior mesenteric vein
